# Controlled beams of shock-frozen, isolated, biological and artificial nanoparticles

**DOI:** 10.1063/4.0000004

**Published:** 2020-04-17

**Authors:** Amit K. Samanta, Muhamed Amin, Armando D. Estillore, Nils Roth, Lena Worbs, Daniel A. Horke, Jochen Küpper

**Affiliations:** 1 Center for Free-Electron Laser Science, Deutsches Elektronen-Synchrotron DESY, Notkestraße 85, 22607 Hamburg, Germany; 2 Department of Physics, Universität Hamburg, Luruper Chaussee 149, 22761 Hamburg, Germany; 3 Center for Ultrafast Imaging, Universität Hamburg, Luruper Chaussee 149, 22761 Hamburg, Germany

## Abstract

X-ray free-electron lasers promise diffractive imaging of single molecules and nanoparticles with atomic spatial resolution. This relies on the averaging of millions of diffraction patterns of identical particles, which should ideally be isolated in the gas phase and preserved in their native structure. Here, we demonstrated that polystyrene nanospheres and *Cydia pomonella* granulovirus can be transferred into the gas phase, isolated, and very quickly shock-frozen, i.e., cooled to 4 K within microseconds in a helium-buffer-gas cell, much faster than state-of-the-art approaches. Nanoparticle beams emerging from the cell were characterized using particle-localization microscopy with light-sheet illumination, which allowed for the full reconstruction of the particle beams, focused to <100 μm, as well as for the determination of particle flux and number density. The experimental results were quantitatively reproduced and rationalized through particle-trajectory simulations. We propose an optimized setup with cooling rates for particles of few-nanometers on nanosecond timescales. The produced beams of shock-frozen isolated nanoparticles provide a breakthrough in sample delivery, e.g., for diffractive imaging and microscopy or low-temperature nanoscience.

## I. INTRODUCTION

Nanometer objects are of extraordinary importance in nature, for example in the complex biological machinery of viruses.^1^ Furthermore, the 21st century has been hailed as the “age of nanotechnology,” with the advent of, e.g., novel nanomaterials, such as quantum-dot light emitting diodes^2^ and nanomedicine.^3^ Understanding the fundamental functionality of these systems requires high-resolution structural information. Recent years have seen phenomenal progress in this area. One pioneering approach to measure direct structural information from isolated nanoparticles is single-particle diffractive imaging (SPI), enabled by the advent of x-ray free-electron lasers (XFELs).^4–6^ This promises the recording of atomically resolved structures from isolated nanoobjects without the need for large, highly ordered crystalline samples.^4,5^ It relies on recording a series of two-dimensional (2D) diffraction images from randomly oriented isolated particles, which can then be assembled *in silico* to a three-dimensional (3D) diffraction volume and the structure can be reconstructed. Since the first demonstration of this approach a decade ago,^7^ several significant steps in experimental procedures^8,9^ and data analysis^10^ have pushed the achievable resolution to below 10 nm.^11^

Another technique for direct structural imaging of nanometer-sized objects is cryo-electron microscopy (CEM), where several recent breakthroughs have enabled single-particle structure determination to sub-nanometer resolution.^12,13^ Unlike SPI, CEM images a single nanoparticle, immobilized and shock-frozen onto a support. This sample preparation using the plunge-freezing approach is a crucial step for CEM success.^14^ However, various issues with the technique have been discussed.^14^

In contrast to CEM, the SPI approach images isolated particles *in vacuo*, i.e., without any mechanical sample support. However, due to its diffraction-before-destruction approach,^4^ it requires the imaging of millions or billions of identical particles to allow the reconstruction of the 3D structure. One of the major challenges for improving the achievable resolution is the reproducibility of the target, i.e., the stream of isolated single particles probed by the XFEL. To date, these experiments have been conducted with room temperature aerosols, in an attempt to keep the biological systems studied under native-like conditions. This approach also leads to a dynamical exploration of the conformational landscape. It demands the collection of a very large dataset for SPI experiments, which then have to be analyzed for structures in terms of conformations and spatial orientations. Eventually, this will also limit the achievable resolution for a given measurement time. Moreover, these experiments are often struggling with limited hit rates and limited availability of time for experiments at XFEL facilities, which represent a major obstacle for the collection of a sufficiently large dataset required for a high-resolution reconstruction.

Here, we propose and demonstrate a novel sample preparation using rapidly shock-frozen beams of, potentially hydrated, isolated nanoparticles. Using a cryogenic buffer-gas cell, nanoparticles were rapidly cooled on a microsecond timescale, sufficiently fast to prevent denaturation, and then extracted into a collimated particle stream in vacuum. The produced high-density beams make an ideal target for SPI experiments and, furthermore, are highly amenable to further control, e.g., by external electric or acoustic fields. The cryogenic-temperature samples will allow one to spatially separate conformers,^16–20^ to strongly align and orient the particles in the laboratory frame,^21,22^ or to produce very high densities through the focusing of the particles with external fields.^23,24^

Our development provides touch-free shock-frozen sub-10 K focused particle beams of artificial and biological nanoparticles. Particles were aerosolized from solution at room temperature using a gas-dynamic virtual nozzle^25,26^ and transported into a cryogenically cooled helium-filled buffer-gas cell, in which isolated nanoparticles were quickly cooled through collisions with the cold helium gas. Buffer-gas cooling is an established technique in atomic and molecular physics,^27^ but had so far not been applied to systems with more than a few tens of atoms.^28,29^ We demonstrate its applicability to shock-freeze polystyrene spheres (PS) of 220 nm and 490 nm diameter, as well as the native occlusion bodies (OBs) of *Cydia pomonella* granulovirus (CpGV) particles with a size of approximately $265\times 265\times 445\;nm^{3}$.^30^ The shock-frozen particles were extracted from the buffer-gas cell and formed a collimated or focused nanoparticle beam. Here, individual particles were detected using single-particle-localization microscopy.^31^ Measured particle distributions for different helium-flow conditions were well reproduced by particle-trajectory simulations, which furthermore allowed us to extract the cooling rates and times.

## II. METHODS

### A. Experimental details

**FIG. 1.**
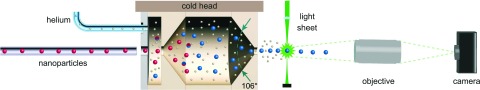
Schematic of the experimental setup. Aerosolized nanoparticles (red spheres) are transported into the cryogenically cooled buffer-gas cell, where they collisionally thermalize (blue spheres) with precooled helium (brown spheres). They exit the cell forming a beam of cold nanoparticles, which is characterized using a single-particle localization microscope. The buffer-gas cell has detachable conical entrance and exit endcaps with a full opening angle of 106°.^15^

A schematic of our experimental setup is shown in Fig. 1. It consists of four main parts: an aerosolization chamber, a differentially pumped transport tube, a cryogenically cooled buffer-gas cell (BGC), and a detection region. Isolated nanoparticles were created by aerosolising aqueous solutions using a gas-dynamic virtual nozzle.^25,26^ We have used polystyrene spheres of 220 nm (Alfa Aesar, 220±17.3 nm) and 490 nm (Molecular Probes, 490 ± 15 nm) with a concentration of $5\times 10^{10}$ particles/ml. CpGV samples were produced following a known protocol.^30^ After leaving the aerosolization chamber, the particles passed through a set of two skimmers ($\emptyset_{1}=0.3$ mm, $\emptyset_{2}=0.5$ mm) placed 2 mm apart. The region between the skimmers was evacuated to remove background gases from the aerosolization processes, e.g., helium and water, to avoid ice formation and clogging of the BGC inlet and outlet. The particle stream then entered a transport tube, with a typical pressure of 10 mbar during operation. The warm isolated nanoparticles were introduced into the buffer-gas cell using a 10 cm long stainless steel capillary with an inside diameter of 800 *μ*m. The complete aerosol generation and transport assembly is attached to the vacuum chamber using a three-dimensional position manipulator, allowing precise alignment of the capillary to the 2 mm buffer-gas cell inlet. During the experiment, the capillary tip was located 7 mm outside the cell entrance aperture. The buffer-gas cell was located in the main vacuum chamber, maintained at a pressure below $10^{-6}$ mbar by a turbomolecular pump (Pfeiffer Vacuum HiPace 2300). It was attached to a two-stage pulse-tube refrigerator (Sumitomo RP082E2) with typical operating temperatures of 29 K and 3.6 K, shielded from thermal radiation by aluminum and copper heat shields attached to the cooling stages. Coconut charcoal attached to the second stage radiation shield provides additional pumping capacity. The buffer-gas cell itself was a hollow copper cylinder (∅=3 cm, 2 cm length) with detachable copper endcaps for both entrance and exit sides. We used conical endcaps with an opening angle of 106°.^15^ Inside the buffer-gas cell, the room-temperature nanoparticles underwent rapid collisional thermalization with the 4 K cold helium gas at typical densities of $∼10^{16}\;{\text{cm}}^{-3}$. The cooled nanoparticles were extracted through an exit aperture of 2 mm diameter into a high vacuum, $p<10^{-6}$ mbar, forming a collimated/focused particle beam,^32^ while the density of the helium gas dropped quickly.^33^ Particles were detected 10 mm after the exit of the cell by particle-localization microscopy based on optical light scattering.^31^ The use of a light sheet to illuminate particles allowed a large-area illumination and hence direct measurement of the entire transverse profile of the particle beam.^34^ The generated particle size distribution was monitored using a commercial differential mobility analyzer (TSI 3786) and a condensation particle counter (TSI 3081).

### B. Simulation details

The experiments were complemented by quantitative simulations of nanoparticles traveling through the apparatus. To model the gas-particle interactions within the buffer-gas cell, we developed a numerical simulation framework capable of calculating the buffer-gas flow field, trajectories of particles in the flow field, and the resulting particle temperatures. The velocities and pressures of the helium flow-field were obtained by solving the Navier–Stokes equation at 4 K using a finite element solver^35^ for different mass flow conditions. Then, using a homebuilt simulation framework, particle trajectories were calculated within the evaluated steady-state flow field according to Stoke's law. A temperature dependent particle-slip-correction factor is required to calculate the drag forces.^36^ As no such correction factor was reported for cryogenic temperatures, we used the known values for air in the range 200–1000 K (Ref. 36) scaled up by a factor of 4 to give consistent results with our experiment at the cryogenic temperature. Due to the low nanoparticle densities, we assumed no effect of the particles on the flow-field and no particle-particle interactions. Numerical integration was performed using the Dormand and Prince Runge–Kutta method dopri5 as provided in scipy.integrate.ode. The flow-field data are linearly interpolated using scipy.interpolate.RegularGridInterpolator.^37^ The particles' phase-space distribution at the inlet of the buffer-gas cell was assumed to be Gaussian, with mean values and standard deviations obtained from simulating particle trajectories in the transport tube and capillary using a cylindrically symmetric model for that part of the setup. Simulations through the buffer-gas cell were performed using both, a two-dimensional (2D) description assuming cylindrical symmetry and a three-dimensional (3D) exact experimental geometry. The latter was deemed necessary because of small deviations of the apparatus from the cylindrical symmetry due to the precooled-helium inlet, see Fig. 1. At high helium flows, this led to a noticeable asymmetry in the produced particle distribution, which was well-reproduced by the 3D simulations, *vide infra*. Initial phase-space distributions of particles at the entrance of the buffer-gas cell were taken from equivalent simulations of the transport system.^32^ The final phase space distribution of the particle beam was collected at a detector placed 10 mm behind the buffer-gas cell outlet.

Nanoparticle temperatures were evaluated by two independent approaches. A collision-based model was used to calculate the temperature drop per helium-particle collision, ensuring conservation of energy and momentum.^27^ This yields the particles' translational temperature, but does not take into account the thermal properties or the internal heat capacity of particles. In the second approach, the heat transfer from the nanoparticle into the buffer gas was estimated by calculating the Nusselt number for forced convection of flow past a single sphere.^38^ The cooling rate, taking into account the heat capacity of the particles, was then estimated according to Newton's law of cooling

$$T(t)=T_{\text{He}}+(T(0)-T_{\text{He}})e^{-hA/C},$$ (1)

with the temperature *T*(*t*) of the particle at time *t*, $T_{\text{He}}=4$ K, the initial temperature of polystyrene T(0)=298 K, the surface area *A* of the nanoparticle, the total heat capacity *C*, which is the specific heat capacity *C_p_* multiplied by the particle mass, and the heat transfer coefficient *h*. The latter was obtained by calculating the Nusselt number *Nu* for a flow past a sphere using the Whitaker formula^38^

$$Nu=2+\left(0.4Re^{1/2}+0.06Re^{2/3}\right)Pr^{0.4}{\left(\frac{\mu_{b}}{\mu_{0}}\right)}^{1/4},$$ (2)

with the Reynolds number *Re*, the Prandtl number *Pr*, and the fluid viscosity $\mu_{b}$ evaluated at the bulk temperature $T_{\text{He}}=4$ K, and the fluid viscosity *μ*_0_ evaluated at the initial surface temperature T(0)=298 K. As the mean free path of the helium gas is larger than the nanoparticle diameter, a rarefied-gas correction was used^39^

$$Nu=\frac{Nu_{0}}{1+3.42\frac{Ma}{\mathrm{RePr}}Nu_{0}},$$ (3)

with the Nusselt number in the continuum regime *Nu*_0_ and the Mach number *Ma*. The heat transfer coefficient *h* was calculated as h=kNu/D with *D* being the diameter of the nanoparticle and *k* the thermal conductivity of helium.

## III. RESULTS AND DISCUSSION

**FIG. 2.**
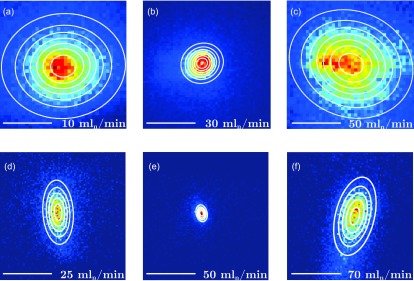
Experimental particle beam profiles of polystyrene spheres. Profiles of the particle beam emerging from the buffer-gas cell for different helium flow rates at the position of the light sheet (a)–(c) for 220 nm and (d)–(f) for 490 nm polystyrene spheres. The scalebars in the left bottom of the figure represent 500 *μ*m, the individual helium gas flows are specified at the bottom right of every panel, and the color coding represent increasing particle flux from blue to red. Contour lines (white) represent 2D Gaussian fits; see the text for details.

**FIG. 3.**
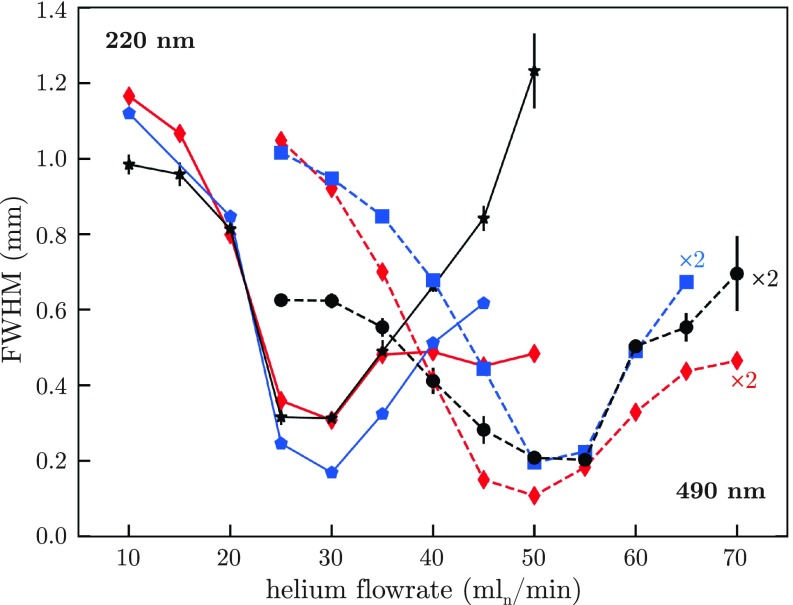
Focusing behavior of polystyrene spheres. Measured particle beam widths for 490 nm (dashed lines) and 220 nm (solid lines) polystyrene spheres as a function of helium flow rate. Black lines represent the experimental data, while red lines are from two-dimensional-axisymmetric and blue lines from three-dimensional simulations, as discussed in the text. For the 490 nm datasets, the widths are scaled by a factor of two to improve visibility of the variation.

Spatial profiles of shock-frozen particles in the detection region are shown in Fig. 2 for 220 nm and 490 nm PS for different helium flow rates. The strong variations of the particle beams for different flow conditions clearly indicate a strong interaction, i.e., many collisions, with the helium gas. For the experimental detector position 10 mm behind the cell outlet, the most collimated particle beam was observed at helium flow-rates of 30 and 50 ml_*n*_/min for 220 nm and 490 nm PS, respectively. From Fig. 2, it is evident that the particle distributions were not spherically symmetric, but elliptical. We attribute this to an asymmetric helium flow-field, caused by the location of our helium inlet at the top of the buffer-gas cell inlet. Despite careful cell design, including a first gas inlet chamber for providing a quasi-axisymmetric flow into the main cell,^15^ at large flow rates, significant asymmetries existed in the gas flow, see Fig. S1. We quantified the size of the particle beam using a two-dimensional (2D) Gaussian, indicated by the contour lines shown in Fig. 2. The measured dependence of the particle beam size on the helium flow is shown in Fig. 3 (black curves). Here, we used the mean of the full width at half maximum (FWHM) of the minor and major axes of the 2D Gaussian to quantify the produced beam size. Individual plots for the major and minor axis for both particle sizes are shown in Fig. S2. For both PS sizes, an increase in helium flow led to a gradual decrease in the particle beam size until it reaches a minimum, i.e., a spatial focus, at the detector. Further increasing the helium flow focused the particle beam further, moving the focus before the detector, which resulted in an again larger beam size at the detector, as evident from the simulated particle beam diameters at different distances from the buffer-gas-cell outlet and for different flow conditions, see Figs. S3–S5. We simulated the measured focusing curves using both, 2D-axisymmetric and three-dimensional (3D) asymmetric, flow-condition models, *vide supra*. Comparisons between the measured and simulated beam widths for 220 nm and 490 nm PS are shown in Fig. 3. All simulations are in very good agreement with the experimental data. This also validated our simulation framework, which thus provides further insight into the fluid-dynamic focusing process. For instance, for 490 nm and 220 nm PS particles and a helium flow of 50 ml_*n*_/min, the simulations yielded particle speeds in the laser-detection region of 16 m/s and 22 m/s, respectively. The simulations also provided the phase-space distributions of the particle beams at different coordinates within the buffer-gas cell, which for three different flow rates are shown in Figs. S4 and S5. These distributions clearly illustrate not only the focusing effect, but also the asymmetry present in the helium flow-field for large flows. While the obtuse angles of the buffer-gas cell significantly reduce the formation of turbulences,^15^ the asymmetry of the flow-field, with some indications of remaining turbulences, led to a significant variation of the particles' transverse velocities, especially at large helium flows. It is also evident from the simulated particle beam diameters at different distances from the buffer-gas-cell outlet, Fig. S3, that at a very low helium flow of 25 ml_*n*_/min, not much focusing occurred and the particle beam was collimated, in contrast to the typical convergence–divergence behavior at higher helium flows, which resembles typical aerodynamic lens systems.^32,40^ At sufficiently high flow rates, the thermalized particles in the buffer-gas cell followed the flow-field and, when traveling through the small orifice, sped up. The large momentum of the particles led to a more ballistic behavior when leaving the buffer-gas cell and thus a significantly lower divergence of the particle beam than that of the gas flow. The exact focusing properties of the nanoparticle beam depended on the particles' momentum and thus its fluid-dynamic properties and mass, as well as the flow-field.^40,41^ Generally, heavier particles require larger gas flows for focusing. The particle transmission also increased with increasing helium flow inside the cell, see Fig. S6. For 220 nm particles, the maximum transmission is achieved for a helium flow of 30–35 ml_*n*_/min at 4 K, with a tenfold increase in transmission compared to the lowest flow rate of 5 ml_*n*_/min. This is attributed to stronger fluid-dynamic forces due to the pressure increase, which efficiently guided the nanoparticles through the buffer-gas cell and minimized losses due to collisions with the walls.^27^ Flow conditions for maximum transmission also coincide well with maximum focusing, yielding a 70 times higher flux at the detector for 30 ml_*n*_/min than for 5 ml_*n*_/min, see Fig. S6. With advanced fluid-dynamic focusing outlets,^32^ beam focusing and particle flux can be improved even further. Moreover, the effect of Brownian motion will be significantly reduced by the 4 K translational temperature compared to previous room-temperature approaches. This is especially important for small particles and thus will strongly improve their focusing and thus the particle densities in single-particle imaging experiments. Our precise flow-field and particle-trajectory simulations allowed us to assess the temperature and cooling rate of particles traveling through the cold buffer-gas cell. The number of collisions with helium required for full thermalization depended on the thermal properties of the particle as well as its size and velocity relative to the gas. In Table I, we provide simulated cooling times to several temperatures and corresponding initial cooling rates, for PS of 10–500 nm diameter as well as for the prototypical protein lysozyme.^4,42^ These were calculated assuming forced convection and Newton's law of cooling and took into account the particles' initial internal energy at room temperature. Full cooling curves, i.e., the modeled temperature drop as a function of time as the particle traveled through the buffer-gas cell and the instantaneous cooling rates, are shown in Figs. S7 and S8, along with results for a simpler momentum-transfer-based cooling model. These simulations show that for particles smaller than ∼50 nm, cooling rates on the order of 10^6^–10^7^ K/s can be achieved. This significantly exceeds the cooling rates for the plunge-freezing approach commonly used in CEM.^43,44^ Furthermore, the simulations show that the cooling rate strongly depends on the initial position of the warm nanoparticle in the cold cell, i.e., on the local helium density, and on the particles' velocity distribution. This provides the way forward toward even faster cooling: Moving the position of the heated inlet capillary into the buffer-gas cell will put the warm particles immediately into regions of higher-helium density. Decoupling the initial-cooling cell from the fluid-dynamic focusing, e.g., in double-cell configurations,^27^ would allow orders of magnitude higher densities of cold helium at the inlet, providing correspondingly faster cooling. This two-cell setup will also enable further control of the fluid-dynamic focusing at the outlet,^32^ enabling strongly increased particle densities in the focus. Besides higher densities and better shock-freezing of biological samples, such improvements and corresponding variability in the experimental parameters would also enable studies of possible effects of the freezing rate on the structure of biological macromolecules.

**FIG. 4.**
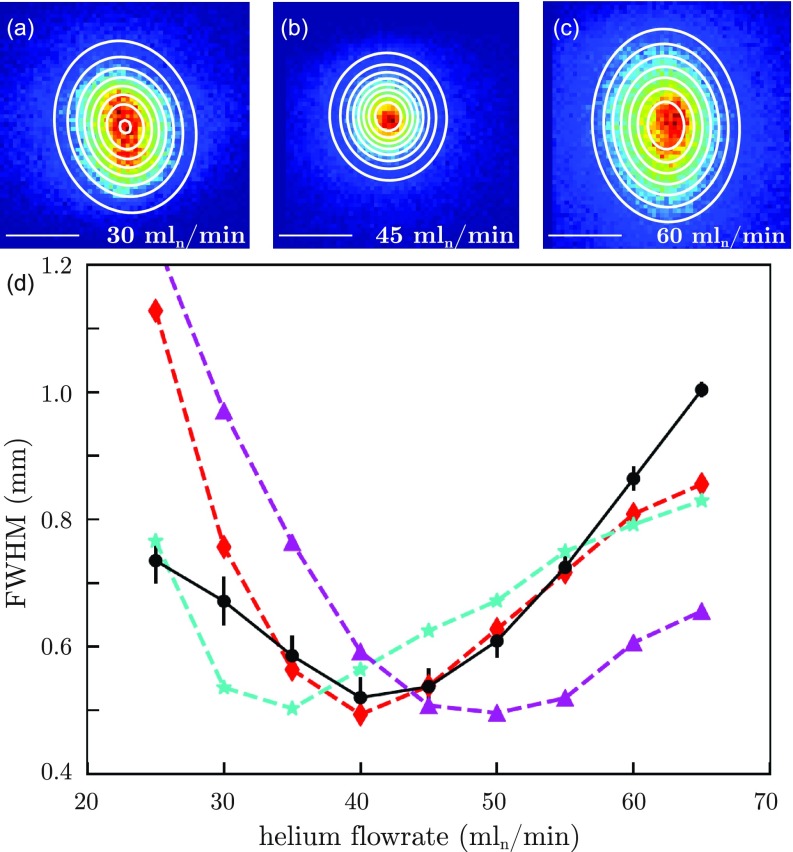
Shock-freezing and focusing granulovirus occlusion bodies. (a)–(c) Experimental particle-beam profiles at the detector position for three different helium flow rates of 30, 45, and 60 ml_*n*_/min, respectively. Scale bars and color codes are the same as Fig. 2. (d) Experimental (black) and simulated (cyan, red, and purple) particle beam widths as a function of helium flow rate. Simulations are shown for three different hydrodynamic diameters: 280 nm (cyan), 320 nm (red), and 360 nm (purple), confirming the expected effective hydrodynamic diameter of 320 nm for CpGV.

**TABLE I.** Cooling rate in the buffer-gas cell for different particle sizes. Calculated cooling rates at a fixed flow rate of 70 ml_*n*_/min and the corresponding cooling times for reaching relevant temperatures, such as the protein glass-transition (200 K), water glass transition (133 K), and liquid nitrogen (77 K) temperatures. The cooling rates have an estimated error of 10%, propagated from the 10% error in the Nusselt number.^39^

	200 K (*μ*s)	133 K (μs)	77 K (μs)	10 K (μs)	Cooling rate (K/s)
500 nm	613	1409	2467	12000	1.8 $\times 10^{5}$
200 nm	224	476	821	3007	4.9 $\times 10^{5}$
50 nm	55	110	185	539	2.2 $\times 10^{6}$
10 nm	12	23	37	103	1.1 $\times 10^{7}$
Lysozyme	6	10	16	40	2.6 $\times 10^{7}$

To study the applicability of our approach to non-spherical biological nanoparticles, we created a cryogenically cooled beam of CpGV. CpGV is readily available commercially as a insecticide alternative to control codling moth populations.^45^ The resulting focusing curves following injection of CpGV into the buffer-gas cell are shown in Fig. 4. As previously observed for PS, the size of the produced particle beam at the detector showed a strong dependence on the helium flow rate, with the narrowest profile and highest density observed for helium flow rates of 40–45 ml_*n*_/min. Our current simulation approach inherently assumes spherical particles. Therefore, to model the CpGV data, we simulated spherical particles of various sizes with a known density of CpGV of 1160 kg/$m^{3}$. Simulations for diameters of 280 nm, 320 nm, and 360 nm are shown in Fig. 4 and the best fit was observed for a particle size of 320 nm as an effective fluid-dynamic diameter for CpGV. This value is in good agreement with the 325 nm obtained as the geometric mean of CpGV's 3D diameters. This indicates that particles inside the buffer-gas cell are freely rotating and no significant flow-alignment effects occur under the experimental conditions of our study. Furthermore, these simulations confirm that also biological particles leave the buffer-gas cell thermalized with the cold helium gas.

## IV. CONCLUSION AND OUTLOOK

We have demonstrated a cryogenic nanoparticle source capable of producing tightly focused beams of shock-frozen aerosolized nanoparticles and its quantitative description. Using a helium buffer-gas cell, isolated room-temperature particles are rapidly cooled, typically reaching liquid nitrogen temperatures within hundreds of microseconds, and quickly thermalizing with the buffer gas at 4 K. The current outlet of the cell acts as a simple fluid-dynamic lens, efficiently extracting particles and forming a focused beam. These beams were characterized through particle-localization microscopy. The cooling and focusing properties can be tuned by varying the helium flow rate and its temperature. A novel numerical simulation infrastructure was setup to provide quantitative simulations of particle trajectories and phase-space distributions, which are in very good agreement with the measurements. These simulations then enabled the extraction of cooling rates and particle temperatures, highlighting the very fast shock-freezing of nanoparticles. Last, but not least, we demonstrated the applicability to non-spherical biological nanoparticles by producing beams of shock-frozen granulovirus particles. Further improvements of the setup will provide orders of magnitude faster cooling rates of the particles as well as better focusing of the emerging beams: The initial cooling can be improved by placing the particle inlet into the buffer-gas cell and through two-cell approaches from small-molecule buffer-gas cooling.^27^ The latter will also allow advanced fluid-dynamic focusing outlets^32^ resulting in strongly increased particle densities in the focus. The demonstrated high-flux beams of shock-frozen nanoparticles will be beneficial to a wide range of experiments in structural biology, nanoscience, and physics, including high-resolution single particle x-ray and electron diffractive-imaging. In particular, our approach, together with control and selection, will overcome the sample variability problem typically encountered in single-particle coherent x-ray diffraction measurements, where millions of particles are needed to create a 3D structure.^46^ Furthermore, the beams of cold isolated particles open up a large tool-box of control methods, originally developed for cold small gas-phase molecules,^17,47^ to these large nanoscale systems. These include the separation of structural isomers or major folding structures^17,18,48^ or molecular alignment approaches that fix molecules in the laboratory frame using optical fields.^21,47,49–51^ Such control would enable the experimental averaging of imaging data over many identical molecules/particles. Furthermore, it provides the prerequisites for future time-resolved studies of ultrafast biochemical dynamics, which require well-defined starting states to controllably and reliably trigger specific dynamic processes of interest. Additionally, the ability to control the particles' final temperature and cooling rate will allow the exploration of the ground-state potential energy landscape and answer important outstanding questions regarding the preservation of native-like conditions upon rapid-freezing. It furthermore enables the direct study of important temperature and size dependent phenomena in artificial nanoparticles, such as extremely large magnetoresistance^52^ or light-induced superconductivity.^53^ Furthermore, it could propel matter-wave interference to new limits.^54^

Our approach enables imaging experiments that bring the benefits of CEM, well-controlled and static sample particles, to single-particle imaging where they can be imaged *in vacuo* without support structures and with ultrafast time-resolution. In turn, combining the very fast cooling enabled by our approach with soft-landing techniques could lead to strong and crucial progress in sample delivery in CEM experiments.

## SUPPLEMENTARY MATERIAL

See the supplementary material for Fig. S1: Helium flow field for different flow conditions in the buffer-gas cell, Fig. S2: Measured dependence of the particle beam size on the helium flow rates for polystyrene spheres, Fig. S3: Simulated focusing behavior at different helium flow rates, Fig. S4: Phase space distributions of a beam of 490 nm PS particles 5 mm before the buffer-gas cell outlet, Fig. S5: Phase space distributions of a beam of 490 nm PS particles 5 mm after the buffer-gas cell outlet, Fig. S6: Experimentally obtained transmission and average flux of particles, Fig. S7: Simulated thermalization times of nanoparticles, and Fig. S8: Rate of cooling as a function of temperature.

## AUTHOR'S CONTRIBUTIONS

The project was conceived by J.K. and coordinated by D.A.H. and J.K. The experiment was designed by A.S., N.R., D.A.H., and J.K.; set up by A.S., A.E., and L.W.; and performed by A.S. and A.E. The data analysis was performed by A.S. and D.A.H. and numerical simulations were performed by M.A., N.R., and A.S.; the results from the theory and experiment were analyzed by A.S., M.A., D.A.H., and J.K. and discussed with all authors. The manuscript was prepared by A.S., D.A.H., and J.K. and discussed with all authors.
